# Seroprevalence of Antibodies against Highly Pathogenic Avian Influenza A (H5N1) Virus among Poultry Workers in Bangladesh, 2009

**DOI:** 10.1371/journal.pone.0073200

**Published:** 2013-09-05

**Authors:** Sharifa Nasreen, Salah Uddin Khan, Eduardo Azziz-Baumgartner, Kathy Hancock, Vic Veguilla, David Wang, Mahmudur Rahman, A. S. M. Alamgir, Katharine Sturm-Ramirez, Emily S. Gurley, Stephen P. Luby, Jacqueline M. Katz, Timothy M. Uyeki

**Affiliations:** 1 Center for Communicable Diseases, International Centre for Diarrhoeal Disease Research, Bangladesh, Dhaka, Bangladesh; 2 Centers for Disease Control and Prevention, Atlanta, Georgia, United States of America; 3 Institute of Epidemiology, Disease Control and Research, Government of Bangladesh, Dhaka, Bangladesh; University of Hong Kong, Hong Kong

## Abstract

We conducted a cross-sectional study in 2009 to determine the seroprevalence and risk factors for highly pathogenic avian influenza A (H5N1) [HPAI H5N1] virus antibodies among poultry workers at farms and live bird markets with confirmed/suspected poultry outbreaks during 2009 in Bangladesh. We tested sera by microneutralization assay using A/Bangladesh/207095/2008 (H5N1; clade 2.2.2) virus with confirmation by horse red blood cell hemagglutination inhibition and H5-specific Western blot assays. We enrolled 212 workers from 87 farms and 210 workers from three live bird markets. One hundred and two farm workers (48%) culled poultry. One hundred and ninety-three farm workers (91%) and 178 market workers (85%) reported direct contact with poultry that died during a laboratory confirmed HPAI H5N1 poultry farm outbreak or market poultry die-offs from suspected HPAI H5N1. Despite exposure to sick poultry, no farm or market poultry workers were seropositive for HPAI H5N1 virus antibodies (95% confidence interval 0–1%).

## Introduction

Highly pathogenic avian influenza (HPAI) A [HPAI H5N1] virus (clade 2.2.2) was first identified among poultry in Bangladesh in March 2007 [1]. As of 29 April 2013, 549 commercial and backyard poultry farms had confirmed HPAI H5N1 outbreaks across 51 out of 64 districts [2]. The first human case of HPAI H5N1 virus infection in Bangladesh was identified in a child during 2008 through routine community surveillance for influenza in Dhaka. A chicken was purchased from the local market and slaughtered in the child’s home before he developed a febrile respiratory illness [3].

Risk factors for avian-to-human transmission of HPAI H5N1 virus include direct or close contact with sick or dead infected poultry, or visiting a live bird market [4–6]. Approximately five million people in Bangladesh are employed in large-scale and small-scale poultry farms [7]. Poultry sellers who handle live poultry, and slaughter, defeather or eviscerate chickens without the use of any personal protective equipment may have occupational exposure to HPAI H5N1 virus.

The frequency or the risk of avian-to-human HPAI H5N1 virus transmission, including risk of clinically mild illness and asymptomatic infection, among poultry workers in Bangladesh is unknown. Understanding the risk of avian-to-human HPAI H5N1 virus transmission from poultry to humans can assist in planning intervention activities that could prevent human infection with HPAI H5N1 virus. We conducted a cross-sectional study during 2009 to determine the seroprevalence of HPAI H5N1 virus antibody and risk factors for HPAI H5N1 virus infection among poultry workers in farms with laboratory confirmed HPAI H5N1 poultry outbreaks or in live bird markets with poultry die-offs from suspected HPAI H5N1 virus infection.

## Methods

### Ethics statement

The study team obtained written informed consent from the workers before enrollment. The institutional review boards at icddr,b, IEDCR and CDC reviewed and approved the research protocol.

### Settings

#### Poultry farms with HPAI H5N1 outbreaks

We conducted this study in poultry farms across Bangladesh that reported HPAI H5N1 outbreaks that were laboratory confirmed by the Department of Livestock Services (DLS), under the Ministry of Fisheries and Livestock, Bangladesh. The DLS coordinated poultry culling in these farms and hired day laborers to help the farm workers to cull the poultry. The DLS supplied personal protective equipment (gown, apron, gloves, cap, goggles, N95 mask and shoe covers) to all persons involved in culling. The Institute of Epidemiology, Disease Control and Research (IEDCR) under the Ministry of Health and Family Welfare, initiated a two-week follow-up evaluation of poultry workers and cullers immediately after the culling. During the daily follow-up IEDCR dispensed post-exposure oseltamivir chemoprophylaxis (75 mg once daily for 7 days) and observed each person for clinical signs and symptoms of influenza-like illness [8].

#### Live bird markets

There were approximately 127 live bird markets (wholesale and retail) in Dhaka where live poultry were sold during 2008–2009 (The Chief Veterinary Officer, DLS, personal communication). Live birds from all over Bangladesh were sold in these markets. Live birds sold in the markets included chickens (layer, broiler and indigenous), ducks, geese, pigeons and quail. Wholesale markets remained open 7 days a week, 24 hours a day, with workers taking shifts depending upon the type of work they did. Wholesale markets had up to a few hundred shops, between one to two thousand workers, and sold thousands of poultry daily. Some of these wholesale markets were also retail markets. Retail markets were open from 6 A.M. to 11 P.M., had approximately 5-100 shops and 15-150 workers, and sold up to a few thousand poultry daily. Some workers came from outside Dhaka city to work for a few hours a day or few days a month in these markets. All shops sold live poultry. Unsold caged poultry remained in the markets for a few days and were mixed with birds from newly arrived flocks. The majority of the workers sold live poultry. Most of these workers also slaughtered, defeathered and eviscerated birds that were sold in the markets. Some workers only slaughtered, defeathered and eviscerated poultry. The DLS initiated surveillance for avian influenza A viruses among poultry in the live bird markets of Dhaka, but the results from this surveillance were not available due to sensitivity of the information.

### Selection of poultry farms and live bird markets

We collected a list of farms from the DLS with confirmed HPAI H5N1 poultry outbreaks that occurred during 29 December 2007 to 22 June 2009. We collected the list of farms where IEDCR followed poultry workers. We also used the daily update of poultry outbreaks from the Ministry of Fisheries and Livestock (http://www.mofl.gov.bd/) and the monthly country report submitted to the World Organization for Animal Health by DLS (http://www.oie.int/downld/AVIAN%20INFLUENZA/A_AI-Asia.htm) to ensure we identified all reported outbreak farms. In total, we identified 131 farms with confirmed HPAI H5N1 outbreaks; 99 (75%) farms with outbreaks during 28 December 2007–18 May 2008, 6 (5%) with outbreaks during July–December 2008 and 26 (20%) with outbreaks during January–July 2009.

We selected three live bird markets: two of which were the largest wholesale and retail markets in Dhaka. We assumed that workers at these markets were more likely to have been exposed to HPAI H5N1 virus-infected poultry than at smaller markets because dealers brought the poultry from all over Bangladesh. We used the presence of ≥5–10% mortality in caged poultry for two consecutive days as a proxy indicator of possible HPAI H5N1 virus circulation among these poultry because we lacked information on laboratory confirmed HPAI H5N1 virus infection among poultry in these markets. Workers from all three markets reported that they experienced this level of poultry mortality sporadically during 2008.

### Sample size estimation and poultry worker enrollment

Assuming the prevalence of HPAI H5N1 virus neutralizing antibody to be 2.5% among poultry workers in Bangladesh, with a confidence level of 95% and 1.5% confidence interval, our estimated study sample size was 416 participants. This was based on a lower estimated seroprevalence of HPAI H5N1 virus antibodies among occupationally exposed workers, ranging from 0% [9–11] to 10% [12]. We estimated that seroprevalence would be greater than 0% because of the high prevalence of HPAI H5N1 viruses circulating among poultry in Bangladesh and likely high exposures of poultry workers to HPAI H5N1 virus-infected poultry. We enrolled poultry workers aged 18 through 59 years who reported having direct and close contact with poultry. Since this study was the first HPAI H5N1 virus antibody seroprevalence study conducted among poultry workers in Bangladesh, the age range of 18 through 59 years was used to maximize specificity for detection of neutralizing antibody to HPAI H5N1 virus using microneutralization assay combined with confirmatory Western blot and horse red blood cell hemagglutination inhibition assays [13]. Although day laborers participated in poultry culling, we did not recruit or enroll any of day laborers because of their short exposure and use of personal protective equipments provided by the DLS. During 19 January-27 July 2009, field teams visited 129 farms and the three markets to recruit, enroll, administer questionnaires and collect blood specimens from the poultry workers. Although we identified 131 farms with a history of laboratory confirmed HPAI H5N1 poultry outbreaks, we could not locate two farms. Forty farms did not have any workers as the farms had shut down because of the outbreaks. Workers were available from 89 farms. Workers who were present on these farms during the outbreaks and present at the time of the field team visit were asked for their written informed consent to participate and enrolled. In the live bird markets we prepared a list of 305 workers who were present from 8: 00 AM to 5: 00 PM and the field teams approached all available workers during this time.

### Questionnaire

Using a pre-tested questionnaire (questionnaire S1 and questionnaire S2), the field team collected information on demographics, poultry exposure, use of personal protective measures and history of respiratory illness around the time of poultry outbreaks. When interviewing market workers, field teams also observed and recorded workers’ use of personal protective measures during poultry handling.

### Blood collection

Medical technologists collected a 10 ml blood specimen from each participant. All blood samples collected within Dhaka were transported in a cold box at 2–8°C to the icddr,b laboratory at the end of each day and were centrifuged to separate serum. Outside Dhaka, serum was separated from blood at the end of each day using a portable centrifuge machine, stored in a cold box at 2–8°C and transported to icddr,b within 48 hours of collection. All sera were split into three aliquots and stored at -70°C at icddr,b. One aliquot was shipped frozen on dry ice to CDC, Influenza Division (Atlanta, GA, USA), for serologic testing.

### Laboratory methods

Sera were tested by the microneutralization assay as previously described using A/Bangladesh/207095/2008 (H5N1; clade 2.2.2) virus and modified to use an initial serum dilution of 1:10 [13,14]. We calculated the geometric mean antibody titers obtained in two or more replicate tests with a starting serum dilution of 1:10. A seropositive result was defined as an HPAI H5N1 virus neutralizing antibody titer ≥40 (equivalent to WHO protocol criteria of ≥80), with confirmation by horse red blood cell hemagglutination inhibition assay using the above virus or an H5-specific Western blot assay using a recombinant H5 (clade 2.2) protein as antigen as per WHO criteria [13,15,16].

### Statistical analyses

We performed Wilcoxon rank-sum to test the equality of median age, duration of smoking for current smokers, duration of employment with poultry and duration of poultry contact during work activities and performed two-sample test of proportions (Z-test) to test the equality of proportions for gender, current smoker, chronic medical conditions, types of poultry handled, different types of poultry contact through work activities, contact with poultry that died from illness, post-exposure oseltamivir chemoprophylaxis use and respiratory illness experienced around poultry outbreak/die offs between farm and market workers. We performed two-sample test of proportions (Z-test) to test the equality of proportions for different poultry exposures through work activities, contact with dead poultry at farms, and participation in culling between male and female farm workers. We performed two-sample t-test to compare the mean age of workers with specific poultry exposures to those without these exposures for farm and market workers. We estimated the relative risk of having an HPAI H5N1 virus neutralizing antibody titer ≥20 using a log linear model. We calculated 95% confidence intervals (CIs) for seroprevalence using binomial distribution.

## Results

### Demographics

**Figure 1:**
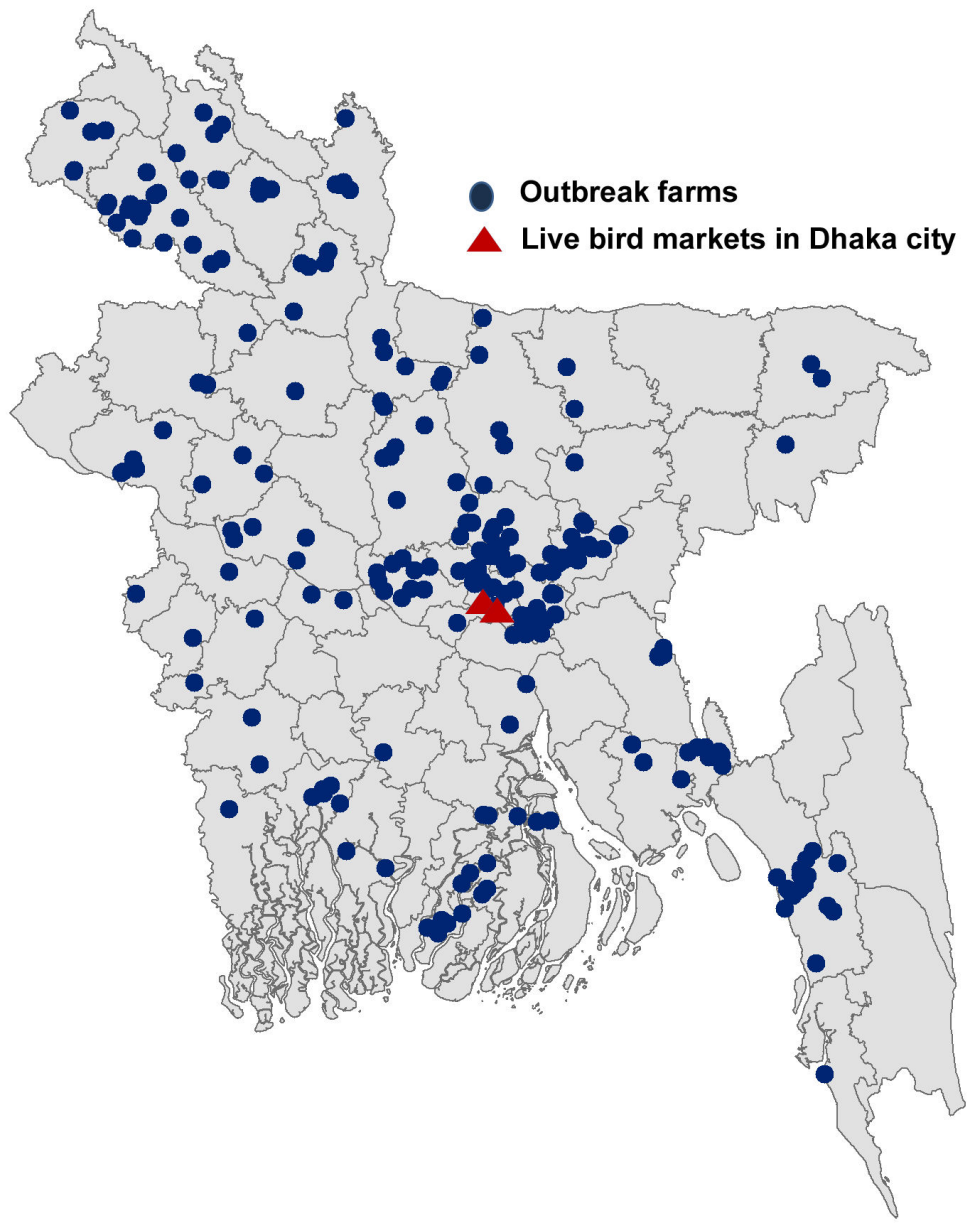
Location of confirmed HPAI H5N1 virus poultry farm outbreaks during December 2007-June 2009 and suspected HPAI H5N1 outbreaks in live bird markets in 2009, Bangladesh.

We enrolled all 212 available workers from 89 farms and 210 workers from 3 live bird markets (Figure 1). None of the available farm workers declined while 40 (16%) of the available market workers refused to participate in the study. Of the farm workers, 66 (31%) were enrolled within 6 months, 4 (2%) were enrolled between 6–12 months, and the remaining 142 (67%) were enrolled between 12–18 months of the onset of poultry die-offs in the farms. The farm workers were older than the market workers (median age: 33 versus 30 years, P=0.019) (Table 1). Median duration of employment involving poultry was longer for market workers compared to farm workers (120 months versus 60 months, P< 0.001) (Table 1).

**Table 1:** Baseline characteristics of poultry workers, Bangladesh-2009.

**Characteristics**	**Participants, n (%)**		***P*-value**
	**Farm worker (N=212)**	**Market worker (N=210)**	
Gender, male	184 (87)	210 (100)	<0.001^*^
Median age in years (IQR)	33 (24–45)	30 (24–38)	0.02^†^
Current smoker	92 (43)	111 (53)	0.05^*^
Median duration of smoking in years (IQR)	8 (4–20)	10 (4–20)	0.7
Chronic medical conditions^‡^	20 (9)	15 (7)	0.4
Median duration of employment with poultry in months (IQR)	60 (36–120)	120 (60–216)	<0.001^†^

^*^ *P*-value for two-sample test of proportion

^†^ *P*-value for Wilcoxon rank-sum test

^‡^Such as lung, liver, kidney, gastro-intestinal and heart diseases, diabetes mellitus and allergies

### Exposures to poultry

Participants reported poultry exposure through one or more routine activities (Table 2). The most common exposures among farm workers occurred during feeding (97%), giving water (83%) and collecting eggs (83%) from poultry, while market workers commonly were exposed during selling (85%), feeding (53%), cleaning water (51%) and feeding trays (48%) (Table 2). One hundred and two (48%) of 212 farm workers reported participating in culling of poultry and 93% reported using personal protective measures during culling (Table 2). One hundred and one (99%) of 102 workers participating in culling reported receiving post-exposure oseltamivir for chemoprophylaxis. Both 91% of farm workers (193/212) and 85% of market workers (178/210) reported direct contact with poultry that died from illness during a confirmed HPAI H5N1poultry outbreak in the farm or suspected poultry die-offs in the market (Table 2).

**Table 2:** Reported occupational exposure to confirmed or suspected HPAI H5N1 virus-infected poultry, use of personal protective measures and respiratory illness among farm and market workers, Bangladesh-2009.

**Variables**	**Participants, n (%)**		***P*-value**
	**Farm worker (N=212)**	**Market worker (N=210)**	
Type of poultry handled (^*^ for market workers)			
Chicken			
Layer	174 (82)	160 (76)	0.1
Broiler	20 (9)	115 (55)	<0.001^†^
Broiler and layer	14 (7)	-	-
Indigenous	1 (0.5)	132 (63)	<0.001^†^
Duck	-	72 (34)	-
Pigeon	-	36 (17)	-
Quail	3 (1)	-	-
Duration of poultry contact during regular activities^‡^			
Hour/day, mean(range)	9 (2–14)	10 (3–18)	<0.001^§^
Days/week, mean (range)	7 (5–7)	7 (3–7)	<0.001^§^
Lived in the farm premises	120 (57)	NA^║^	-
Contact with poultry through regular activities^*‡^			
Sell poultry	-	179 (85)	-
Feed poultry	206 (97)	112 (53)	<0.001^†^
Give water	176 (83)	-	-
Collect egg	176 (83)	-	-
Clean feeding tray	161 (76)	100 (48)	<0.001^†^
Clean water tray	168 (80)	106 (51)	<0.001^†^
Clean stall/faeces	130 (61)	79 (38)	<0.001^†^
Slaughter poultry	81 (38)	82 (39)	0.8
Defeather	47 (22)	62 (30)	0.08
Eviscerate	47 (22)	64 (31)	0.05
Vaccinate poultry (vaccine other than avian influenza)	46 (22)	-	-
Collect or transport faeces	-	1 (0.5)	-
Contact with poultry that died from illness^‡^	193 (91)	178 (85)	0.048^†^
Participated in culling	102 (48)	NA^║^	-
Handled sick poultry during culling (n=102)	96 (94)	-	-
Used personal protective measures during culling (n=102)	95 (93)	-	-
Mask	95 (100)	-	-
Gloves	92 (97)	-	-
Overall/dress	91 (96)	-	-
Washed hands after handling poultry	89 (94)	-	-
Cap	88 (93)	-	-
Eye protection/goggles	78 (82)	-	-
Received post-exposure oseltamivir chemoprophylaxis^¶^	196 (93)	0 (0)	<0.001^†^
For <7 days	9 (5)	-	-
For 7 days	181 (92)	-	-
For >7 days	6 (3)	-	-
Visit to a live bird market around^†^the time of poultry outbreak	26 (12)	NA^║^	-
Reported personal protective measures taken during regular activities^*‡^	110 (52)	Not asked	-
Sprayed disinfectants	90 (82)	-	-
Washed hands after working with poultry	80 (73)	-	-
Wore masks	73 (66)	-	-
Changed clothes	37 (34)	-	-
Wore gloves	30 (27)	-	-
Wore boots/shoes	18 (16)	-	-
Wore goggles	7 (6)	-	-
Frequency of using personal protective measures during regular activities^‡^	n=110	NA^║^	-
Always	67 (61)	-	-
Sometimes (<50% time)	26 (24)	-	-
Most of the times (>50% time)	17 (16)	-	-
Respiratory illness around^**^ poultry outbreak/die off	37 (17)	59 (28)	0.009^†^

^*^Multiple responses. Percentages do not sum up to 100.

^†^ *P*-value for two-sample test of proportions

^‡^For farm workers, one week before poultry outbreaks to end of culling; for market workers, one week before to one week after the poultry die-offs

^§^ *P*-value for Wilcoxon rank-sum test

^║^Not applicable

^¶^Oseltamivir was distributed to the farm workers by the government during culling

^**^7 before to 14 days after

Among the farm workers, a higher proportion of female workers reported cleaning poultry feeding trays (93% [26/28] vs. 73% [135/184], p=0.025), defeathered poultry (43% [12/28] vs. 19% [35/184], p=0.005), and eviscerated poultry (36% [10/28] vs. 13% [24/184], p=0.002), compared to male workers, respectively. A higher proportion of male workers reported participated in poultry culling compared to female workers (54% [100/184] vs. 7% [2/28], p=0.000). Farm workers who participated in poultry culling were younger compared to those who did not participate in culling (mean age: 33 vs. 37 years, p=0.02). Market workers who fed poultry (mean age: 30 vs. 34 years, p=0.02), cleaned feeding trays (mean age: 30 vs. 34 years, p=0.006), cleaned water trays (mean age: 30 vs. 34 years, p=0.02), cleaned poultry feces (mean age: 30 vs. 33 years, p=0.02), slaughtered poultry (mean age: 30 vs. 33 years, p=0.01), defeathered poultry (mean age: 29 vs. 33 years, p=0.02), eviscerated poultry (mean age: 29 vs. 33 years, p=0.009) and touched dead poultry (mean age: 31 vs. 33 years, p=0.04) were younger than workers without these poultry exposures. One hundred and ten (52%) of 212 farm workers reported using one or more protective measures during daily poultry care (Table 2). When asked, 99% of market workers responded that they washed their hands while working in the market. The use of protective measures, such as wearing masks, gloves or handwashing, however, were not observed by study field staff during data collection.

### Seroprevalence of HPAI H5N1 virus antibody

The median time interval between date of onset of poultry die-offs on the farms and collection of blood specimens from farm workers was 444 days, range (22–543). No farm or live bird market poultry workers tested seropositive for clade 2.2.2 HPAI H5N1 virus neutralizing antibodies (95% confidence interval 0–1%). One market worker had a titer of 57 by MN assay, but had negative results by confirmatory horse red blood cell hemagglutination inhibition and western blot assays. An additional seven market workers (3%) had titers of ≥20 and <40. One market worker that had an HPAI H5N1 virus neutralizing antibody titer of 20 by MN assay reported feverishness, measured temperature ≥100.4°F, cough, runny nose, bodyache and headache around the time of poultry die-offs. All titers for farm workers were <20. As none of the workers met the criteria for seropositivity but some of the market workers had a titer of ≥20, we conducted a post-hoc analysis to estimate the relative risk of having a neutralizing antibody titer of ≥20 among the market workers. The relative risk was higher among workers who reported that they slaughtered (RR 11.0 [7/82 (9%) vs. 1/128 (1%), 95% CI 1.4–87.1]), defeathered (RR 7.2 [6/62 (10%) vs. 2/148 (1%), 95% CI 1.5–34.5]) or eviscerated (RR 6.8 [6/64 (9%) vs. 2/146 (1%), 95% CI 1.4–33.0]) poultry compared to workers who did not conduct those activities in the markets. Workers with an HPAI H5N1 virus neutralizing antibody titer ≥20 were younger compared to workers with an antibody titer <20 (Table 3).

**Table 3:** Characteristics of live bird market workers with HPAI H5N1 virus neutralizing antibody titers <20 and ≥20 by microneutralization (MN) assay, Bangladesh-2009.

**Characteristics**	**<20 neutralizing antibody titer by MN assay (N=202) n, (%)**	**≥20 neutralizing antibody titer by MN assay (N=8) n, (%)**
Median age in years (IQR)	30 (24–38)	24 (21–33)
Smoker	111 (55)	0 (0)
Chronic medical conditions	15 (7)	0 (0)
Median duration of employment with poultry in months (IQR)	120 (60–216)	48 (33–90)
Contact with poultry in the market		
Fed poultry	107 (53)	5 (63)
Cleaned feeding tray	95 (47)	5 (63)
Cleaned water tray	101 (50)	5 (63)
Cleaned cages/feces	76 (38)	3 (38)
Slaughtered	75 (37)	7 (88)
Defeathered	56 (28)	6 (75)
Eviscerated	58 (29)	6 (75)
Collected/transported feces	1 (0)	0 (0)
Contact with dead poultry in the market	173 (86)	5 (63)

## Discussion

Despite extensive direct and close exposure to poultry at both farms and markets, we did not find any serologic evidence of HPAI H5N1 clade 2.2.2 virus infection among the serum samples collected from either farm or market poultry workers during the study period. Our results are similar to other HPAI H5N1 virus antibody seroprevalence studies conducted among poultry workers such as in Nigeria (clade 2.2 virus) [10], Indonesia (clade 2.1 virus) [17] and Vietnam (clade 1 virus) [18] where no serologically confirmed infections were observed. Other studies in China have reported 0.2–3% seroprevalence among poultry workers [9,19,20] with concurrent detection of HPAI H5N1 virus in poultry and at live bird markets, using either hemagglutination inhibition assay alone or with confirmation by microneutralization assays. The highest seroprevalence in poultry workers (estimated at 10%) was reported from a 1997 study in Hong Kong of clade 0 HPAI H5N1 virus neutralizing antibodies [12].

None of the individual poultry workers met the criteria for seropositivity in our study which were those recommended by WHO [15] and were developed to maximize both sensitivity and specificity for detection of strain-specific anti-H5 antibodies as evidence of HPAI H5N1 virus infection. Eight market workers did have low HPAI H5N1 virus antibody titers by microneutralization assay; for seven workers, titers were only 2-fold above the limit of detection for this assay and were not confirmed by any other method. Slaughtering, defeathering and evisceration of poultry were associated with an HPAI H5N1 virus antibody titer of ≥20 among market workers in our study. Other studies have not found an association between low HPAI H5N1 virus antibody titers and poultry worker occupational risk factors [21].

It is difficult to interpret these low levels of HPAI H5N1 virus neutralizing antibodies in a cross-sectional study. First, low titers may indicate cross reactive antibodies from a previous influenza A virus infection with a different subtype rather than the presence of HPAI H5N1 virus-specific neutralizing antibodies [11,13]. Single or multiple-clade H5N1 influenza vaccines can generate cross-clade neutralizing antibodies in humans and mice [22,23]. Nevertheless, only HPAI H5N1 clade 2.2.2 viruses were identified among domestic poultry in Bangladesh during 2007–2009 [1]. Second, these low titers could potentially reflect a limited neutralizing antibody response in some individuals with HPAI H5N1 virus infection. One study in Vietnam of persons with serologic evidence of clinically mild or asymptomatic HPAI H5N1 virus infection reported relatively lower neutralizing antibody titers compared with severely ill cases [24]. Third, low titers may reflect past HPAI H5N1 virus infection with declining neutralizing antibody titers over time to when serum was sampled, to levels below our defined cut-off titer defining a seropositive result [24,25].

We could not ascertain the actual time interval between exposure to affected poultry and collection of blood samples for the market workers. These workers probably experienced multiple or on-going exposures to HPAI H5N1 virus-infected poultry during 2008–2009 [26,27]. The long intervals between poultry farm outbreaks and subsequent collection of blood specimens from the poultry workers may have reduced our ability to detect HPAI H5N1 virus antibodies. Collection of serum closer to the time of exposure to confirmed HPAI H5N1 virus outbreaks among poultry and at multiple time points would have enabled us to better assess the kinetics of the HPAI H5N1 virus neutralizing antibody response over time among farm and market workers. Other methods to assess the cellular immune response such as measuring H5N1-specific T-cell responses might help identify prior HPAI H5N1 virus infections that resulted in asymptomatic or mild illness [28]. Exposure to HPAI H5N1 virus in poultry workers can potentially include self-inoculation of mucous membranes (including conjunctivae) of the respiratory tract after direct contact with poultry or surfaces contaminated with feces, internal organ tissues, or poultry blood; or through inhalation of large or small droplets that are aerosolized such as those generated through slaughtering, defeathering, or evisceration [29].

Most of the farm workers used some personal protective equipment during culling and more than half reported using protective measures during daily poultry care, which may have reduced their exposure to HPAI H5N1 virus. Although all participants tested seronegative, market workers may have had greater exposure to sick or dead HPAI H5N1 virus-infected poultry compared to farm workers because of unprotected contact, higher frequency of contact and longer occupational exposure. Avian influenza surveillance in live bird markets in Dhaka has detected HPAI H5N1 viruses in multiple cloacal, fecal or oropharyngeal poultry samples and in environmental samples which suggests ongoing transmission of HPAI H5N1 viruses since 2008 [26,27,30]. HPAI H5N1 virus has also been detected from similar environmental samples from live bird markets in Indonesia [31].

Considering the ongoing exposure of market workers, safe poultry handling practices during these high-risk activities should be promoted, such as designated slaughtering areas in the markets with accessible running water and soap, and actively promoting use of personal protective measures, including respiratory protection [32]. Other technically appropriate interventions such as slaughtering poultry in a bag could minimize direct contact and reduce aerosolization of HPAI virus. In the longer term, when financially viable, designated slaughtering plants could be introduced. Mathematical models have demonstrated that weekly market rest days could be effective in reducing transmission of HPAI H5N1 virus [33]. Monthly rest days implemented in Hong Kong were associated with reduced detection of H9N2 virus in chickens in retail live poultry markets [34]. Compulsory weekly rest days have been recently initiated in the live bird markets in the capital city, Dhaka. These measures could be effective in reducing market worker exposures to all avian influenza A viruses, including HPAI H5N1 virus.

Our findings need to be interpreted in light of several limitations. To select live birds markets for this study, we used the criterion of reported ≥5–10% daily poultry die-offs for two consecutive days among caged poultry without laboratory testing as a proxy for HPAI H5N1 virus infection of poultry. The identified market die-offs coincided with confirmed HPAI H5N1 poultry outbreaks in farms nationwide and surveillance had detected HPAI H5N1 virus in poultry and environmental samples collected from live bird markets in Dhaka [26,27,30]. Thus workers in these markets likely had HPAI H5N1 virus exposures. During an outbreak of human cases of HPAI H5N1 virus in Hong Kong in 1997, exposure to poultry with >10% mortality was highly associated with H5 seropositivity among poultry workers [12]. Additionally, poultry wholesalers at a live bird market, the source of poultry for the first human case of HPAI H5N1 virus infection in Bangladesh, also reported 5–10% die-offs among caged chickens at that time [35]. Another limitation was that poultry workers were interviewed a few weeks to several months after the exposure and the long recall period might have affected their ability to accurately recall the types of exposures they may have had during the outbreaks. Since the poultry die offs were major events in the farm workers lives, however, we expect that they remembered the events. The farm workers may have given socially acceptable answers (courtesy bias) during self-reporting of protective measure use, therefore the reported use of protective measures is likely overestimated. Similarly, the market workers may have over-reported their hand washing.

We did not find serologic evidence of HPAI H5N1 virus infection among farm or market poultry workers during 2009, consistent with a seroprevalence of ≤1%. Nevertheless, HPAI H5N1 viruses continue to infect poultry in Bangladesh, and since January 2011, there have been over 162 reported poultry outbreaks, a three-fold increase compared to the same period in 2010 [36]. The Food and Agriculture Organization classifies Bangladesh as one of the six endemic countries for HPAI H5N1 virus in poultry [37]. During 2011, two new clades of HPAI H5N1 virus have been detected in Bangladesh: clade 2.3.2 that caused extensive crow die-offs, and clade 2.3.4 identified among poultry [38,39]. Pediatric cases of illness because of HPAI H5N1 virus infection and a case of illness with low pathogenic avian influenza A (H9N2) virus infection were also identified in 2011 [40]. HPAI H5N1 viral RNA was identified by real-time reverse transcription polymerase chain reaction from nasal and throat swabs of three poultry workers with mild upper respiratory tract illness in Dhaka in 2012 though live bird market surveillance [41,42]. These cases together with the first fatal case of HPAI H5N1 virus infection in 2013 indicates an on-going public health risk of transmission of HPAI H5N1 virus and other avian influenza A viruses to humans in Bangladesh [43].

Our study demonstrated that the occupational risk of HPAI H5N1 clade 2.2.2 virus infection among poultry workers appeared to be low in 2009, but the risk that this infrequent event represents to global public health remains substantial [44]. In Bangladesh, with a population density of 964/square kilometers for 142 million population and around 50% chickens being reared in backyards, the impact of even low frequency of poultry-to-human transmission of avian influenza A viruses, including HPAI H5N1 virus, will be greater than in sparsely populated countries [7,45]. Our study findings can serve as a baseline for future HPAI H5N1 virus serosurveys in Bangladesh. Serial collection of sera, for example, sampling at intervals <6 months, will allow better interpretation of HPAI H5N1 virus antibody titers. Continued close monitoring is warranted for possible avian influenza A virus infections among poultry workers in the country.

## Supporting Information

Questionnaire S1 **Questionnaire for farm poultry workers.**
(DOC)

Questionnaire S2 **Questionaire for live bird market poultry workers.**
(DOC)
