# Supplementary material for: Seroprevalence of Antibodies against Highly Pathogenic Avian Influenza A (H5N1) Virus among Poultry Workers in Bangladesh, 2009
Source: PLoS One. 2013 Sep 5;8(9):e73200. doi: 10.1371/journal.pone.0073200 (PMC3764173; doi:10.1371/journal.pone.0073200)
Supplement: Questionnaire S1 — Questionnaire for farm poultry workers. (DOC) [file pone.0073200.s001.doc]

**Supplemental information:**

**Seroprevalence of antibodies against highly pathogenic avian influenza A (H5N1) virus among poultry workers in Bangladesh, 2009**

Sharifa Nasreen^1^, Salah Uddin Khan^1^, Eduardo Azziz-Baumgartner^2^, Kathy Hancock^2^, Vic Veguilla^2^, David Wang^2^, Mahmudur Rahman^3^, ASM Alamgir^3^, Katharine Sturm-Ramirez^1, 2^, Emily S. Gurley^1^, Stephen P. Luby^1,2^, Jacqueline M. Katz^2^, Timothy M. Uyeki^2^

^1^ icddr,b, Dhaka, Bangladesh

^2^ Centers for Disease Control and Prevention (CDC), Atlanta, Georgia, USA

^3^ Institute of Epidemiology, Disease Control and Research (IEDCR), Government of Bangladesh, Dhaka, Bangladesh

*Corresponding author:

Sharifa Nasreen, MBBS, MPH

Centre for Communicable Disease,

icddr,b, Bangladesh

Phone: + (1) 352-213-8591

Email: drsharifa74@gmail.com, drsharifa@icddrb.org

**S1: Questionnaire for poultry farm workers**

| **International Centre for Diarrhoeal Disease Research, Bangladesh**  **Assessing prevalence and risk factors of mild/asymptomatic H5N1 infections among persons exposed to H5N1 infected poultry**  **Poultry farm workers questionnaire** |
| --- |

| Interviewer : 1  2  3  Date of interview : ___/___/___ (Date/Month/Year)   | ID |  |  |  |  |  |  |  |  |  |  | | --- | --- | --- | --- | --- | --- | --- | --- | --- | --- | --- |   Interview start time: __.__ AM/PM End time: __.__ AM/PM  Place of interview : ____________________________  Type of farm : (1) Commercial large scale  (2) Commercial small scale  Type of poultry : Broiler: (1) Yes (2) No  Layer: (1) Yes (2) No  Quail: (1) Yes (2) No  Indigenous: (1) Yes (2) No  Date of culling in the farm: ___/___/___ (Date/Month/Year)  Number of poultry culled in the farm: ______________________ |
| --- | --- | --- | --- | --- | --- | --- | --- | --- | --- | --- | --- |

| *Section A: Socio-demographic information*  *I am going to start with questions about your socio-demographic conditions* |
| --- |

*A1. What is your name? _____________________________*

*A2. Sex of the respondent (to be observed)*

*Male 1*

*Female 2*

*A3. How old are you?*

*___ ___ years (completed)*

*DK 88*

*NR 99*

*A4. How many years of schooling have you completed?*

*___ ___ years*

*DK 88*

*NR 99*

*A5. What is your average monthly family/household expenditure (in taka)?*

*1,000-5,000 Tk 1*

*5,001-10,000 Tk 2*

*10,001-15,000 Tk 3*

*15,001-20,000 Tk 4*

*20,001-25,000 Tk 5*

*25,001-30,000 Tk 6*

*30,001-40,000 Tk 7*

*40,001-50,000 Tk 8*

*>50,000 Tk 9*

*DK 88*

*NR 99*

| ***Section B: Information on contact with infected poultry during the outbreak*** |
| --- |

*B1. How long have you worked in a poultry farm (in total)?*

*__ __ years __ __ months*

*B2. How long have you been working in this farm (in total)?*

*__ __ years __ __ months*

***Now I am going to ask you questions about your work during the outbreak that is one week before the start of poultry deaths through the end of the culling***

*B3. How many chicken died from disease during the outbreak before culling in the farm?*

*___________________*

*B4. During that time that is the time between seven days before the start of poultry deaths and culling how often did you work in the poultry shed on average?*

*____ (hours per day)*

*____ (days per week)*

*B5. Which of the following jobs did you do around poultry then?* ***Mention all the options*** *(circle ‘Yes’ or ‘No’ for each. If ‘Yes’, write down the frequency)*

|  |  | ***Yes*** | ***Frequency/day*** | ***No*** |
| --- | --- | --- | --- | --- |
| *a.* | *Feed poultry* | *1* |  | *2* |
| *b.* | *Give water to poultry* | *1* |  | *2* |
| *c.* | *Collect egg* | *1* |  | *2* |
| *d.* | *Clean poultry feeding tray* | *1* |  | *2* |
| *e.* | *Clean water tray* | *1* |  | *2* |
| *f.* | *Clean poultry stall/faeces* | *1* |  | *2* |
| *g.* | *Slaughter poultry* | *1* |  | *2* |
| *h.* | *Defeather* | *1* |  | *2* |
| *i.* | *Eviscerate* | *1* |  | *2* |
| *j.* | *Take poultry to the market* | *1* |  | *2* |
| *k.* | *Take eggs to the market* | *1* |  | *2* |
| *l.* | *Vaccinate poultry* | *1* |  | *2* |
| *m* | *Hatch eggs* | *1* |  | *2* |

*B6. Did you touch any dead poultry that died in the farm during that time?*

*Yes 1*

*No 2* ***(Go to Q B7)***

*B6a. If yes, what did you do with the dead chicken? (circle ‘Mentioned’, ‘Not mentioned’ or ‘INAP’ for each)*

|  |  | ***Mentioned*** | ***Not mentioned*** | ***INAP*** |
| --- | --- | --- | --- | --- |
| *a.* | *Eviscerate* | *1* | *2* | *7* |
| *b.* | *Bury* | *1* | *2* | *7* |
| *c.* | *Burn* | *1* | *2* | *7* |
| *d.* | *Throw in garbage pile/ponds/drains* | *1* | *2* | *7* |
| *e.* | *Ate* | *1* | *2* | *7* |
| *f.* | *Other (specify)*  *________________________* | *1* | *2* | *7* |

*B7. Did you take any specific measure while you worked in the farm at that time?*

*Yes 1*

*No 2* ***(Go to Q B8)***

*B7a. If yes, what measure(s) did you take? (circle ‘Mentioned’ , ‘Not mentioned’ or ‘INAP’ for each)*

|  |  | ***Mentioned*** | ***Not mentioned*** | ***INAP*** |
| --- | --- | --- | --- | --- |
| *a.* | *Change dress* | *1* | *2* | *7* |
| *b.* | *Wear mask* | *1* | *2* | *7* |
| *c.* | *Wear gloves* | *1* | *2* | *7* |
| *d.* | *Wash hand after working with poultry* | *1* | *2* | *7* |
| *e.* | *Eye protection/goggles* | *1* | *2* | *7* |
| *f.* | *Spray disinfectant* | *1* | *2* | *7* |
| *g.* | *Other (specify)*  *________________________* | *1* | *2* | *7* |

*B7b. How often did you take the measure?*

*Sometimes 1*

*Most of the time 2*

*Always 3*

*INAP 7*

*B8. Did you take part in culling?*

*Yes 1*

*No 2* ***(Go to Q B9)***

*B8a. What was your role in culling? (circle ‘Mentioned’, ‘Not mentioned’ or ‘INAP’ for each)*

|  |  | ***Mentioned*** | ***Not mentioned*** | ***INAP*** |
| --- | --- | --- | --- | --- |
| *a.* | *Handle sick poultry* | *1* | *2* | *7* |
| *b.* | *Handle apparently healthy poultry* | *1* | *2* | *7* |
| *c.* | *Cervical dislocation* | *1* | *2* | *7* |
| *d.* | *Putting culled chicken in bags* | *1* | *2* | *7* |
| *e.* | *Bury chicken* | *1* | *2* | *7* |
| *f.* | *Other (specify)*  *_____________________* | *1* | *2* | *7* |

*B8b. Did you take any specific measure during your participation in culling?*

*Yes 1*

*No 2* ***(Go to Q B9)***

*INAP 7*

*B8b(i). If yes, what measure did you take? (circle ‘Mentioned’, ‘Not mentioned’ or ‘INAP’ for each)*

|  |  | ***Mentioned*** | ***Not mentioned*** | ***INAP*** |
| --- | --- | --- | --- | --- |
| *a.* | *Wear dress/overall* | *1* | *2* | *7* |
| *b.* | *Cap* | *1* | *2* | *7* |
| *c.* | *Wear mask* | *1* | *2* | *7* |
| *d.* | *Wear gloves* | *1* | *2* | *7* |
| *e.* | *Wash hand after working with poultry* | *1* | *2* | *7* |
| *f.* | *Eye protection/goggles* | *1* | *2* | *7* |
| *g.* | *Other (specify)*  *________________________* | *1* | *2* | *7* |

*B9. Did you take any medicine (Tab. Oseltamivir) given by the government during/after culling?*

*Yes 1*

*No 2* ***(Go to QB10)***

*B9a. If yes, how many times did you take the medicine daily?*

*____ times*

*INAP 7*

*B9b. How long did you take the medicine?*

*____ days*

*INAP 77*

*B10. Did you live in the premises?*

*Yes 1*

*No 2* ***(Go to Q B11)***

*B10a. How far was your dwelling place from the poultry shed?*

*______________ feet* ***(Go to Q B14 )***

*INAP 77*

*B11. Did you have any backyard poultry in your home at that time?*

*Yes 1*

*No 2*

*INAP 7*

*B12. Did you come into contact with any sick or dead poultry in your home?*

*Yes 1*

*No 2*

*INAP 7*

*B13. Did you slaughter any poultry at home at that time?*

*Yes 1*

*No 2*

*INAP 7*

*B14. Did you come into contact with any backyard poultry in the community then?*

*Yes 1*

*No 2*

B15. Did you visit any live bird market during that time?

Yes 1

No 2 ***(Go to Q B16)***

B15a. If yes, how many times did you go?

_______________ times

INAP 77

B16. Did you visit any poultry feed selling shop at that time?

Yes 1

No 2 ***(Go to Section C)***

B16a. If yes, how many times did you go?

_______________ times

INAP 77

| **Section C: Information on febrile or respiratory illness during the outbreak**  **Now I am going to ask you questions about any respiratory illness you had during the time between seven days before the onset of poultry death and 14 days after culling** |
| --- |

*C1. Did you develop any of the following symptoms during that time period?* (circle ’Yes’ or ‘No’ for each)

|  |  | **Yes** | **No** |  |
| --- | --- | --- | --- | --- |
| a. | Feverishness | 1 | 2 |
| b. | Measured temperature ≥ 100.4 ^0^F | 1 | 2 |
| c. | Cough | 1 | 2 |
| d. | Sore throat | 1 | 2 |
| e. | Runny nose | 1 | 2 |
| f. | Body ache | 1 | 2 |
| g. | Headache | 1 | 2 | ***(Go to SectionD)*** |
| h. | Red or watery eyes | 1 | 2 |  |
| i. | Vomiting | 1 | 2 |
| j. | Diarrhoea | 1 | 2 |
| k. | Fever, cough & respiratory distress (ILI) | 1 | 2 |
| l. | Difficulty breathing or shortness of breath, or | 1 | 2 |
| m. | Breathing fast? | 1 | 2 |

C2. Did you go to any pharmacy/kabiraj/doctor for the symptoms you had then?

Yes 1

No 2

INAP 7

C3. Did you take any medication/traditional treatment/herbal remedies other than the ones given to you by the government?

Yes 1

No 2 **(Go to Q C4)**

INAP 7

C3a. If yes, what medication/traditional treatment/herbal remedy did you take?

___________________________________________

Don’t know 8

INAP 7

C4. Were you hospitalized for any of respiratory symptoms at that time??

Yes 1

No 2 ***(Go to Section D)***

INAP 7

C4a. If yes, where were you admitted to?

__________________________

INAP 7

C4b. How many days did you stay in the hospital?

_________ days

INAP 77

| **Section D: Information on smoking, chronic illness and chronic medication** |
| --- |

D1. Do you smoke?

Yes 1

No 2 ***(Go to Q D2)***

D1a. If yes, how long have you been smoking?

__ __ years

INAP 77

D1b. How many cigarettes/bidi did you smoke yesterday?

___ ___ cigarettes/bidi

INAP 77

D2. Has a doctor ever told you that you have lung disease?

Yes 1

No 2 ***(Go to Q D3)***

*D2a. If yes, what disease do you have? (circle ‘Mentioned’, ‘Not mentioned’ or ‘INAP’ for each* . If ‘Mentioned’, write down duration of illness)

|  |  | **Mentioned** | **Duration of illness (Months, Days)** | **Not mentioned** | **INAP** |
| --- | --- | --- | --- | --- | --- |
| a. | Asthma | 1 |  | 2 | 7 |
| b. | Emphysema | 1 |  | 2 | 7 |
| c. | Other (specify)  _______________ | 1 |  | 2 | 7 |

D3. Do you have any other chronic illness?

Yes 1

No 2 ***(Go to Q D4)***

*D3a. If yes, what illness(es) do you have? [probe: any other] (circle ‘Mentioned’, ‘Not mentioned’ or ‘INAP’ for each* . If ‘Mentioned’, write down duration of illness)

|  |  | **Mentioned** | **Duration of illness (Months, Days)** | **Not mentioned** | **INAP** |
| --- | --- | --- | --- | --- | --- |
| a. | Liver disease | 1 |  | 2 | 7 |
| b. | kidney disease | 1 |  | 2 | 7 |
| c. | Gastro-intestinal disease | 1 |  | 2 | 7 |
| d. | Heart disease | 1 |  | 2 | 7 |
| e. | Diabetes | 1 |  | 2 | 7 |
| f. | Allergy | 1 |  | 2 | 7 |
| g. | Other (specify)  _______________ | 1 |  | 2 | 7 |

D4. Do you take any medicine regularly/daily?

Yes 1

No 2 ***(End of interview for male respondent)***

## D4a. If yes, what is/are the name(s) of the medicine(s) and the dose, frequency and duration? (For the interviewer: If the respondent has the medicine with him/her, please see the medicine and record both generic and trade names)

|  | Name of the medicine | Dose | Frequency | Duration (month) | INAP |
| --- | --- | --- | --- | --- | --- |
| a. |  |  |  |  | 7 |
| b. |  |  |  |  | 7 |
| c. |  |  |  |  | 7 |
| d. |  |  |  |  | 7 |
| e. |  |  |  |  | 7 |
| f. |  |  |  |  | 7 |
| g. |  |  |  |  | 7 |

## (End of interview for male respondents. For female respondents ask Q D5)

## D5. Are you currently married? (For female respondents only)

Yes 1

No 2 ***(End of interview)***

INAP 7

D5a. If yes, are you pregnant now?

Yes 1

No 2 ***(End of interview)***

INAP 7

D5b. What is your duration of pregnancy?

__________ months

INAP 77

## Thank you for your cooperation and participation in the survey

Survey completed: (1) YES (2) NO

If no, reason for incomplete survey:

1. Refused to complete
2. Postponed
3. Other (Specify)____________
