# Supplementary material for: Seroprevalence of Antibodies against Highly Pathogenic Avian Influenza A (H5N1) Virus among Poultry Workers in Bangladesh, 2009
Source: PLoS One. 2013 Sep 5;8(9):e73200. doi: 10.1371/journal.pone.0073200 (PMC3764173; doi:10.1371/journal.pone.0073200)
Supplement: Questionnaire S2 — Questionaire for live bird market poultry workers. (DOC) [file pone.0073200.s002.doc]

**Supplemental information:**

**Seroprevalence of antibodies against highly pathogenic avian influenza A (H5N1) virus among poultry workers in Bangladesh, 2009**

Sharifa Nasreen^1^, Salah Uddin Khan^1^, Eduardo Azziz-Baumgartner^2^, Kathy Hancock^2^, Vic Veguilla^2^, David Wang^2^, Mahmudur Rahman^3^, ASM Alamgir^3^, Katharine Sturm-Ramirez^1, 2^, Emily S. Gurley^1^, Stephen P. Luby^1,2^, Jacqueline M. Katz^2^, Timothy M. Uyeki^2^

^1^ icddr,b, Dhaka, Bangladesh

^2^ Centers for Disease Control and Prevention (CDC), Atlanta, Georgia, USA

^3^ Institute of Epidemiology, Disease Control and Research (IEDCR), Government of Bangladesh, Dhaka, Bangladesh

*Corresponding author:

Sharifa Nasreen, MBBS, MPH

Centre for Communicable Disease,

icddr,b, Bangladesh

Phone: + (1) 352-213-8591

Email: drsharifa74@gmail.com, drsharifa@icddrb.org

**S2: Questionnaire for live bird market poultry workers**

| **International Centre for Diarrhoeal Disease Research, Bangladesh**  **Assessing prevalence and risk factors of mild/asymptomatic H5N1 infections among persons exposed to H5N1 infected poultry**  **Poultry market workers questionnaire** |
| --- |

| Interviewer : 1  2  Date of interview : ___/___/___ (Date/Month/Year)   | ID |  |  |  |  |  |  |  |  |  |  | | --- | --- | --- | --- | --- | --- | --- | --- | --- | --- | --- |   Interview start time: __.__ AM/PM End time: __.__ AM/PM  Place of interview : ____________________________  Sell duck : (1) Yes  (2) No  Sell pigeon : (1) Yes  (2) No  Type of poultry : Broiler: (1) Yes (2) No  Cock: (1) Yes (2) No  Indigenous: (1) yes (2) No  Type of cage : Wire cage: (1) Yes (2) No  Bamboo cage: (1) Yes (2) No  Plastic cage: (1) Yes (2) No |
| --- | --- | --- | --- | --- | --- | --- | --- | --- | --- | --- | --- |
| Section A: Socio-demographic information I am going to start with questions about your socio-demographic conditions |

A1. What is your name? _____________________________

A2. Sex of the respondent (*to be observed*)

Male 1

Female 2

A3. How old are you?

___ ___ years (completed)

DK 88

NR 99

A4. How many years of schooling have you completed?

___ ___ years

DK 88

NR 99

A5. What is your average monthly family/household expenditure (in taka)?

1,000-5,000 Tk 1

5,001-10,000 Tk 2

10,001-15,000 Tk 3

15,001-20,000 Tk 4

20,001-25,000 Tk 5

25,001-30,000 Tk 6

30,001-40,000 Tk 7

40,001-50,000 Tk 8

>50,000 Tk 9

DK 88

NR 99

| **Section B: Information on contact with poultry during the outbreak** |
| --- |

B1. How long have you worked in a live poultry market (in total)?

__ __ years __ __ months

B2. How long have you been working in this poultry market (in total)?

__ __ years __ __ months

**Now I am going to ask you questions about your work during one week before and after the time when there was 10% or more die offs among caged poultry in 2 consecutive days**

B3. How often did you work at the market on average then?

____ (hours per day)

____ (days per week)

B4. Which of the following jobs did you do around poultry? ***Mention all the options*** (*circle ‘Yes’ or ‘No’ for each. If ‘Yes’, write down the frequency*)

|  |  | **Yes** | **Frequency/day** | **No** |
| --- | --- | --- | --- | --- |
| a. | Feed poultry | 1 |  | 2 |
| b. | Clean feeding tray | 1 |  | 2 |
| c. | Clean water container | 1 |  | 2 |
| d. | Clean poultry cage/faeces | 1 |  | 2 |
| e. | Slaughter poultry | 1 |  | 2 |
| f. | Defeather | 1 |  | 2 |
| g. | Eviscerate | 1 |  | 2 |
| h. | Collect or transport faeces | 1 |  | 2 |

B5. Did you touch any dead poultry in the market at that time?

Yes 1

No 2 ***(Go to Q B6)***

B5a. What did you do with the dead chicken at that time? (*circle ‘Mentioned’ , ‘Not mentioned’ or ‘INAP’ for each*)

|  |  | **Mentioned** | **Not mentioned** | **INAP** |
| --- | --- | --- | --- | --- |
| a. | Threw them in the dustbin | 1 | 2 | 7 |
| b. | Sold them | 1 | 2 | 7 |
| c. | Dress/defeather them | 1 | 2 | 7 |
| d. | Ate them | 1 | 2 | 7 |
| e. | Other (specify)  ________________________ | 1 | 2 | 7 |

B6. Did the government distribute any medicine in the market at that time?

Yes 1

No 2 ***(Go to Q B7)***

B6a. Did you take the medicine given to you by the government?

Yes 1

No 2 ***(Go to Q B7)***

INAP 7

B6b. If yes, how many times did you take the medicine daily?

______ times

INAP 7

B6c. How long did you take the medicine?

______ days

INAP 77

B7. Did you come into contact with any backyard poultry in the community then?

Yes 1

No 2

B8. Did you have any backyard poultry in your home at that time?

Yes 1

No 2

B9. Did you come into contact with any sick or dead poultry in your home?

Yes 1

No 2

B10. Did you slaughter any poultry at home at that time?

Yes 1

No 2

B11. Do you have water supply in the market?

Yes 1

No 2 **(G*o to Q B8*)**

B11a. Do you wash your hands while you work in the market?

Yes 1

No 2 **(G*o to Q B8*)**

B11b. If yes, how many times did you wash your hands yesterday?

________ times

INAP 777

B11c. When did you wash your hands?

___________________________________________

INAP 7

B11d. What did you wash your hands with?

Water 1

Water and soap 2

Other (specify)________________________ 3

INAP 7

*B12. Use of any protective measure by the respondent.* (interviewer to observe & circle ‘Yes’ or ‘No’ for each)

|  |  | **Yes** | **No** |
| --- | --- | --- | --- |
| a. | Wearing mask | 1 | 2 |
| b. | Wearing gloves | 1 | 2 |
| c. | Other (specify) _______________________________ | 1 | 2 |

| **Section C: Information on febrile or respiratory illness**  Now I am going to ask you questions about any respiratory illness you had **during the time between seven days before** **the time when there was 10% or more die offs among caged poultry in 2 consecutive days and fourteen days thereafter** |
| --- |

*C1. Did you develop any of the following symptoms during that time period?* (circle ’Yes’ or ‘No’ for each)

|  |  | **Yes** | **No** |  |
| --- | --- | --- | --- | --- |
| a. | Feverishness | 1 | 2 |
| b. | Measured temperature ≥ 100.4 ^0^F | 1 | 2 |
| c. | Cough | 1 | 2 |
| d. | Sore throat | 1 | 2 |
| e. | Runny nose | 1 | 2 |
| f. | Body ache | 1 | 2 |
| g. | Headache | 1 | 2 | ***(Go to SectionD)*** |
| h. | Red or watery eyes | 1 | 2 |  |
| i. | Vomiting | 1 | 2 |
| j. | Diarrhoea | 1 | 2 |
| k. | Fever, cough & respiratory distress (ILI) | 1 | 2 |
| l. | Difficulty breathing or shortness of breath, or | 1 | 2 |
| m. | Breathing fast? | 1 | 2 |

C2. Did you go to any pharmacy/kabiraj/doctor for the symptoms you had then?

Yes 1

No 2

INAP 7

C3. Did you take any medication/traditional treatment/herbal remedies other than the ones given to you by the government?

Yes 1

No 2 ***(Go to Q C4)***

C3a. If yes, what medication/traditional treatment/herbal remedy did you take?

___________________________________________

Don’t know 8

INAP 7

C4. Were you hospitalized for any of respiratory symptoms at that time??

Yes 1

No 2 ***(Go to Section D)***

INAP 7

C4a. If yes, where were you admitted to?

__________________________

INAP 7

C4b. How many days did you stay in the hospital?

_________ days

INAP 77

| **Section D: Information on smoking, chronic illness and chronic medication** |
| --- |

D1. Do you smoke?

Yes 1

No 2 ***(Go to Q D2)***

D1a. If yes, how long have you been smoking?

__ __ years

INAP 77

D1b. How many cigarettes/bidi did you smoke yesterday?

___ ___ cigarettes/bidi

INAP 77

D2. Has a doctor ever told you that you have lung disease?

Yes 1

No 2 ***(Go to Q D3)***

*D2a. If yes, what disease do you have? (circle ‘Mentioned’, ‘Not mentioned’ or ‘INAP’ for each* . If ‘Mentioned’write down duration of illness)

|  |  | **Mentioned** | **Duration of illness (Months, Days)** | **Not mentioned** | **INAP** |
| --- | --- | --- | --- | --- | --- |
| a. | Asthma | 1 |  | 2 | 7 |
| b. | Emphysema | 1 |  | 2 | 7 |
| c. | Other (specify)  _______________ | 1 |  | 2 | 7 |

D3. Do you have any other chronic illness?

Yes 1

No 2 ***(Go to Q D4)***

D3a. If yes, what illness(es) do you have? *[probe: any other] (circle Mentioned’, ‘Not mentioned’ or ‘INAP’ for each . If ‘Mentioned’,, write down duration of illness)*

|  |  | **Mentioned** | **Duration of illness (Months, Days)** | **Not mentioned** | **INAP** |
| --- | --- | --- | --- | --- | --- |
| a. | Liver disease | 1 |  | 2 | 77 |
| b. | kidney disease | 1 |  | 2 | 77 |
| c. | Gastro-intestinal disease | 1 |  | 2 | 77 |
| d. | Heart disease | 1 |  | 2 | 77 |
| e. | Diabetes | 1 |  | 2 | 77 |
| f. | Allergy | 1 |  | 2 | 77 |
| g. | Other (specify)  _______________ | 1 |  | 2 | 77 |

D4. Do you take any medicine regularly/daily?

Yes 1

No 2 ***(End of interview for male respondent)***

## D4a. If yes, what is/are the name(s) of the medicine(s) and the dose, frequency and duration? (For the interviewer: If the respondent has the medicine with him/her, please see the medicine and record both generic and trade names)

|  | Name of the medicine | Dose | Frequency | Duration (month) | INAP |
| --- | --- | --- | --- | --- | --- |
| a. |  |  |  |  | 77 |
| b. |  |  |  |  | 77 |
| c. |  |  |  |  | 77 |
| d. |  |  |  |  | 77 |
| e. |  |  |  |  | 77 |
| f. |  |  |  |  | 77 |
| g. |  |  |  |  | 77 |

## (End of interview for male respondents. For female respondents ask Q D5)

##

## D5. Are you currently married? (For female respondents only)

Yes 1

No 2 ***(End of interview)***

INAP 7

D5a. If yes, are you pregnant now?

Yes 1

No 2 ***(End of interview)***

INAP 7

D5b. What is your duration of pregnancy?

__________ months

INAP 77

## Thank you for your cooperation and participation in the survey

Survey completed: (1) YES (2) NO

If no, reason for incomplete survey:

(1) Refused to complete

(2) Postponed

(3) Other (Specify)____________
